# TRI: Corroding Its Original Intent?

**DOI:** 10.1289/ehp.114-a420

**Published:** 2006-07

**Authors:** Nancy Bazilchuk

If knowledge is power, as the proverb goes, then the EPA’s Toxics Release Inventory (TRI) is a powerful tool indeed. Firefighters and first responders used this nearly 20-year-old public database of toxic chemical emissions to identify potential contamination hot spots after the floods of Hurricane Katrina. Residents have used it to find out what kinds of pollutants are being emitted by nearby industries. Investment companies use it to evaluate whether or not to purchase a company’s stocks. Even the Internal Revenue Service uses it to collect a pollution tax from companies that release ozone-damaging chlorofluorocarbons.

Given the TRI’s extensive use, it should come as no surprise that an EPA proposal to streamline TRI regulations for the 23,000-plus facilities that report under the law has proved highly controversial. The EPA’s plan must be reported, a move that critics say would affect the value of the TRI database for the public at large. But proponents argue that the cost savings that businesses would realize from the relief in paperwork would justify any loss of data.

The arguments matter, because the power of the TRI lies in the information it provides. Authorized by the 1986 Emergency Planning and Community Right-to-Know Act, the TRI doesn’t limit emissions of the more than 650 chemicals it now covers, but merely requires that they be reported by the companies that manufacture, use, or process them. However, when residents find out what is discharged by industries in their neighborhoods, they can and have used the facts to force change. Companies have altered their practices when managers see their facilities top the list for particular chemical discharges. In fact, the myriad of uses of the TRI, and its success in influencing business practices, has surprised both supporters and opponents of the original law [see “Now That You Know,” *EHP* 105:38–43 (1997)].

Between 1998 and 2004, the latest year for which data are available, the industries and federal facilities that report TRI data have voluntarily cut total on- and offsite disposal and other releases of TRI chemicals to the air, water, and land by 45%, or some 3 billion pounds. Since 1988, industries have cut releases of the 299 chemicals covered by the original law by nearly 60%. Because the TRI is so different from traditional end-of-pipe regulatory programs, which put limits on how much pollution can be released, it has drawn widespread praise. “Any program that the States, the Sierra Club and Monsanto can all praise is no doubt a true environmental success story,” wrote 12 state attorneys general in their comments on the proposed TRI changes.

## 117,000 Comments and Counting

The proposed changes would increase from 500 pounds to 5,000 pounds the threshold at which facilities would be allowed to use a brief certification form (Form A) instead of a detailed reporting form (Form R) to report on their toxic chemical waste. This threshold is based on the amount of chemical wastes handled by the facility, not the amount released to the environment. Form R requires a complete accounting of a chemical’s fate—the amount on the site; the amount released to the land, air, or water as emissions; the amount recycled or burned for energy recovery or destruction; and the amount shipped from the plant for treatment or disposal. In contrast, Form A simply certifies that a toxic chemical was used at the facility in at least the regulatory threshold amount, but provides no other details.

The EPA’s plan also contains changes regarding a special subset of 20 chemicals and chemical compounds including mercury, lead, and polycyclic aromatic compounds. Previously, none of these “persistent, bioaccumulative, and toxic” (PBT) chemicals could be reported on Form A. Under the new rule, however, a company may file Form A for PBTs if 500 pounds or less is recycled, used for energy recovery, or treated for destruction. If any amount is released or emitted, however, the company must still use the detailed form. Furthermore, dioxins must still always be reported on the detailed form.

In a separate filing, the EPA notified Congress that it is considering changing the frequency of TRI reporting from yearly to every other year. Even though there has been considerable response to this third proposal, there has been little substantive debate. Federal law requires the EPA to warn Congress a year before beginning rule making on TRI reporting frequency, so the agency is still developing the details for this proposal.

When the EPA’s proposed threshold and PBT changes were published in the 4 October 2005 issue of the *Federal Register*, they unleashed a flood of responses—some 70,000 responses by the 13 January 2006 deadline for public comments. Even after the deadline passed, the response didn’t stop; more than 117,000 comments have been filed with the federal agency to date.

Twelve state attorneys general have called on the EPA to abandon the proposal, and a half-dozen U.S. senators and more than 50 U.S. representatives have also written the agency to question the assumptions of the plan. Recalling the TRI’s genesis in the aftermath of the 1984 Bhopal industrial disaster, Representatives Stephen Lynch (D–MA), Henry Waxman (D–CA), and Dennis Kucinich (D–OH) wrote that the plan “is particularly troubling” in view of a recent petrochemical plant explosion in China that ultimately polluted the drinking water supply for millions of people. The congressmen noted that the EPA’s own analysis showed that allowing industries to use the higher threshold of 5,000 pounds for Form A would allow companies nationwide to release a total of 246,092 pounds of benzene—without reporting the release.

Industry and small business community representatives have countered, however, that the EPA’s proposals meet the intent of the law while saving companies time and money (the TRI already has a small business exemption that allows facilities with fewer than 10 employees—including farms, dry cleaners, and others—to completely skip reporting and data collection). The U.S. Small Business Administration’s Office of Advocacy has been among the most vocal proponents for the changes, arguing that the expanded use of Form A is exactly the kind of incentive that will encourage good waste management.

“The current program does not reward the best environmental performers,” says Kevin Bromberg, assistant chief counsel for environmental policy at the Office of Advocacy. “Under the current system, if you run a facility with perfect chemical management techniques and discharge no highly toxic chemicals, you must still fill out the long Form R. Small businesses that are top environmental performers should be rewarded through less paperwork—the short Form A.”

## Saving Money, Same Data?

A change in reporting thresholds clearly changes the amount of detail available from the TRI; the question is how this change affects the utility of the inventory. For example, the EPA has stated that none of the detailed data now reported for 26 chemicals or chemical classes (such as chromium compounds) would be available under the proposed 5,000-pound limit for non-PBT chemicals. Most of the chemicals for which detailed reporting would be lost are pesticides.

But the EPA claims that Form A reports will remain meaningful because the public will still know that the chemical is present at a facility at levels under the proposed thresholds. “The Form A certifications for these chemicals will provide a range by which waste management quantities and practices may be estimated,” the agency wrote in its proposal.

All told, the EPA estimates that the two threshold changes for Form A would save companies a combined total of about 164,000 hours a year and about $7.4 million in filing costs. The EPA’s economic analysis estimates the annual savings at the facility level for each form avoided is approximately $430 for each non-PBT chemical and $790 for each PBT chemical—or between $2 and $4 per day. This savings would come at a loss of detailed information on more than 12,000 releases and disposals of chemicals around the country, which total 14 million pounds of non-PBT chemicals released to the environment—just 0.34% of the total amount released. Given the PBT chemical exception, however, the EPA proposal permits no loss of such information for releases of those chemicals into the environment.

These savings free up environmental managers to focus on solving problems instead of filling out forms, according to Jeff Gunnulfsen, manager of government relations for the Synthetic Organic Chemical Manufacturers Association, a trade group that supports the changes. “Most of our members may have one regulatory person handling many, many issues such as hazardous waste, TRI, air issues, safety, and FDA, so any burden reduction may help them focus on more pressing matters,” Gunnulfsen says.

Still, official comments filed by several companies suggest that not everyone in the business world thinks the changes will save time or money. Under the law, companies must still track the same information and make the same calculations, even if they end up filing the short form. The company must be able to demonstrate to the EPA, if ever called upon, that they know their forms to be correct.

Indeed, in comments submitted in response to the *Federal Register* notice, Mark Herwig of GE Corporate Environmental Programs wrote, “An analysis of TRI data from 2003 suggests that EPA’s estimated burden reduction resulting from the proposed rule could be overstated by over 50% for all facilities. . . . There are several areas of EPA’s burden analysis that need improvement to accurately characterize TRI reporting burden.” According to a fact sheet compiled by OMB Watch, a nonprofit government watchdog group, many other corporations have expressed similar feelings.

Sean Moulton, director of federal information policy for OMB Watch, says communities lose even if just a small percentage of the total data is lost. For example, because mining and electric utilities report extremely large emissions to the TRI, “they swamp everything,” Moulton says. “In comparison to national totals, releases in Delaware may look small. But if you live in Delaware and are looking for what might affect me and my family, then Delaware is huge.” He adds that many of the chemicals tracked under the TRI—such as arsenic and benzene—are dangerous even in small quantities. So focusing strictly on the relative low number of pounds lost may be a poor measure of the situation.

Mike Flynn, director of the EPA’s Office of Information Analysis and Access within the Office of Environmental Information, which oversees the TRI, says the effect of the changes on communities is an issue the agency takes very seriously.

“The goal is to provide information for communities—that is an important central tenet,” Flynn says. But 99% of the data would still be available, he adds, and data losses would be offset by the “clear benefits in providing incentives for these companies to cut their emissions more. This is one of the issues where we have to find the right balance.”

## State Program Effects

Some states have reacted strongly to the EPA proposal, partly because their pollution prevention and monitoring programs rely on the data provided by facilities for the TRI.

For example, in Washington state, if the 5,000-pound threshold is implemented for non-PBT chemicals, 40% of all chemicals now filed on Form R could be reported on Form A, which would include a loss of detail about the fate of 46,000 pounds of carcinogens, says Idell Hansen, TRI coordinator for the Washington State Department of Ecology. “We will only have the name of the chemical and the location of the facility, and we’ll lose all ability to track that chemical,” she says. “Under the proposed rule, we’d lose all information on eight of the top forty facilities with the greatest relative risk based on 2002 [TRI] data,” including data on some of the highest-risk chemicals such as methyl isocyanate—the chemical behind the Bhopal incident.

An analysis by the nonprofit National Environmental Trust showed that roughly 900 zip codes nationwide—10% of those that are home to a TRI reporting facility—would lose all numerical toxic emissions data. The New York State Attorney General’s office explored the impacts of this loss on 45,000 residents in Tonawanda, New York, a Lake Erie community surrounded by several industrial facilities. According to that analysis, changed thresholds would mean that this one community would be subject to unreported releases of 8,100 pounds of neurotoxic chemicals and 3,100 pounds of chemicals that cause respiratory problems, among other releases.

Jessica Emond, an EPA spokeswoman, says it is important to realize that even if a chemical release is not reported to the TRI, the release is almost always regulated by other environmental laws that protect air and water quality (although Moulton points out these limits frequently apply to only a single medium, such as just water or just air, leaving a loophole for releases to other media). “The EPA sets a high bar for companies,” Emond says. “Even with proposed changes, this doesn’t affect the amount of chemicals that a company would be allowed to release under state and federal laws.”

The EPA’s timetable calls for finalizing its proposed rule changes by December 2006. However, congressional action before then might preempt the agency’s rule making. Three U.S. senators have asked the Government Accountability Office to examine the EPA’s proposal. Additionally, in mid-May the House of Representatives approved an amendment to the Interior Appropriations Bill that would prevent the EPA from spending money to finalize the proposal until October 2008. The fate of that amendment will be decided in conference committee later this year.

## Figures and Tables

**Figure:**
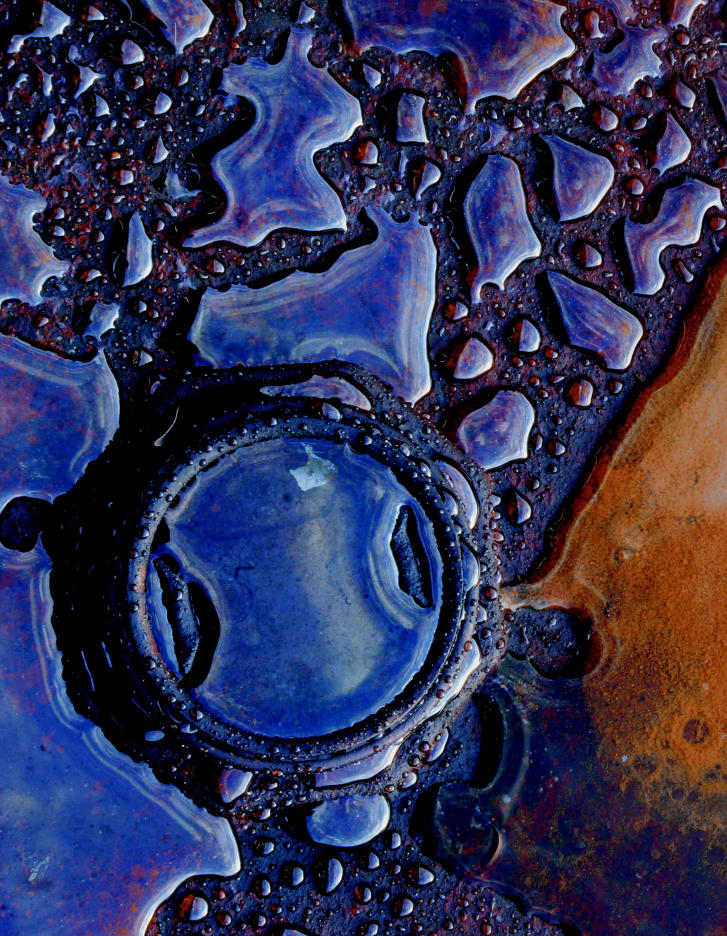


**Figure:**
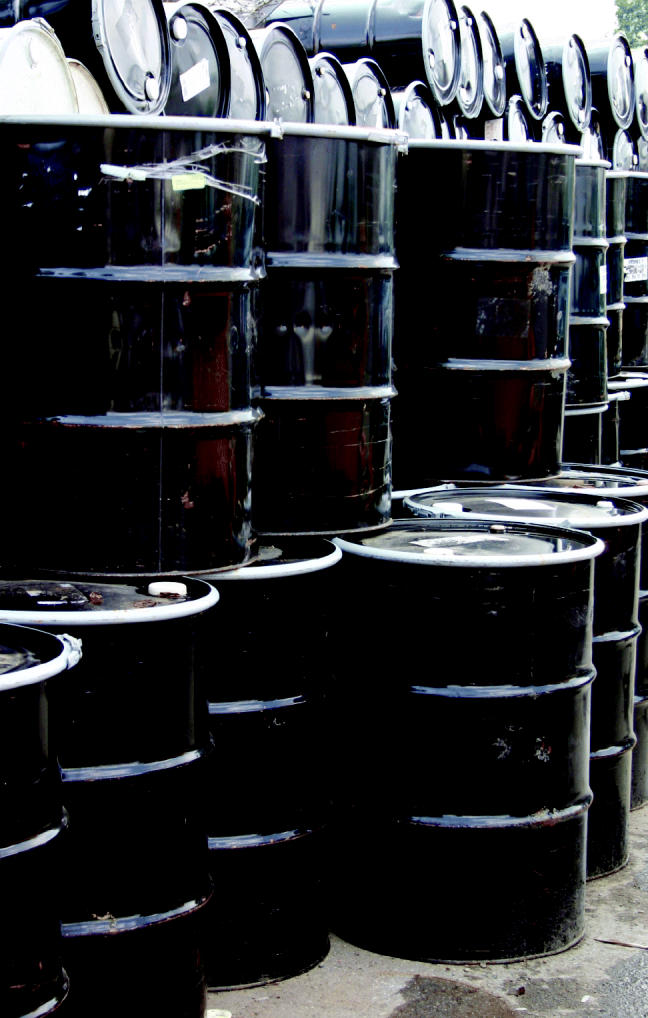
Toxic tradeoff? Changes to the TRI would mean less paperwork for companies but also less information for the public.

